# Stable and dynamic gene expression patterns over diurnal and developmental timescales in *Arabidopsis thaliana*

**DOI:** 10.1111/nph.70023

**Published:** 2025-03-20

**Authors:** Ethan J. Redmond, James Ronald, Seth J. Davis, Daphne Ezer

**Affiliations:** ^1^ Department of Biology University of York Wentworth Way, Heslington York YO10 5DD UK

**Keywords:** *Arabidopsis thaliana*, circadian clock, photoperiodic pathway, plant development senescence, SnRK1

## Abstract

- Developmental processes are known to be circadian‐regulated in plants. For instance, the circadian clock regulates genes involved in the photoperiodic flowering pathway and the initiation of leaf senescence. Furthermore, signals that entrain the circadian clock, such as energy availability, are known to vary in strength over plant development. However, diel oscillations of the Arabidopsis transcriptome have typically been measured in seedlings.
- We collected RNA sequencing (RNA‐seq) data from Arabidopsis leaves over developmental and diel timescales, concurrently: every 4 h d^−1^, on three separate days after a synchronised vegetative‐to‐reproductive transition. Gene expression varied more over the developmental timescale than on the diel timescale, including genes related to a key energy sensor: the sucrose nonfermenting‐1‐related protein kinase complex.
- Moreover, regulatory targets of core clock genes displayed changes in rhythmicity and amplitude of expression over development. Cell‐type‐specific expression showed diel patterns that varied in amplitude, but not phase, over development. Some previously identified reverse transcription quantitative polymerase chain reaction housekeeping genes display undesirable levels of variation over both timescales. We identify which common reverse transcription quantitative polymerase chain reaction housekeeping genes are most stable across developmental and diel timescales.
- In summary, we establish the patterns of circadian transcriptional regulation over plant development, demonstrating how diel patterns of expression change over developmental timescales.

## Introduction

Due to their sessile nature within a cyclical environment, plants have evolved an internal timekeeper called the circadian clock. It consists of a core interlocking loop of transcriptional regulators, whose components are mostly conserved across embryophytes (Petersen *et al*., 2022; Wang *et al*., 2024). Circadian clocks confer fitness to plants by allowing them to coordinate responses to photoperiod, light quality, temperature, and other environmental cues (Dodd *et al*., 2005; Atamian *et al*., 2016; Rubin *et al*., 2017; Xu *et al*., 2022). This coordination takes place through widespread transcriptional control of the plant transcriptome, among other methods of regulation (Nagel *et al*., 2015; Ezer *et al*., 2017; Hayama *et al*., 2017; Romanowski *et al*., 2020; Xiong *et al*., 2022). Estimates of the proportion of the Arabidopsis and wheat transcriptomes that are controlled by the clock range from 30% to 50% (Covington *et al*., 2008; Romanowski *et al*., 2020; Rees *et al*., 2022).

Large‐scale circadian RNA sequencing (RNA‐seq) experiments are typically performed in seedlings or juvenile plants, due to the ease of performing timeseries experiments under different entrainment and free‐running conditions. Yet, many of the clock genes have fundamental roles in the timing of developmental processes (Inoue *et al*., 2018; Wang *et al*., 2024). A key example of this is the evening complex (EC), a tripartite protein complex containing *EARLY FLOWERING 3* and *4* (*ELF3* and *ELF4*) and *LUX ARRHYTHMO* (*LUX*) (Nusinow *et al*., 2011; Herrero *et al*., 2012). The EC, in combination with GIGANTEA (GI), acts upstream of the photoperiodic flowering pathway in Arabidopsis and rice (Fowler *et al*., 1999; Park *et al*., 1999; Sawa & Kay, 2011; Andrade *et al*., 2022). Leaf senescence occurs concurrently with the vegetative‐to‐reproductive transition (Redmond *et al*., 2024) and is also regulated by the circadian clock. Mutations in clock genes, including *ELF3*, *PSEUDO‐RESPONSE REGULATOR9* (*PRR9*), and *CIRCADIAN‐CLOCK ASSOCIATED1* (*CCA1*) have all been shown to affect the onset of leaf senescence (Sakuraba *et al*., 2014; Kim *et al*., 2018; Song *et al*., 2018). This emphasises the need to study circadian rhythms in adult plants when the relevant developmental transitions are occurring.

Moreover, developmentally associated processes exhibit diel or diurnal patterns, meaning that they have an oscillating expression over a 24‐h period. These patterns begin at the earliest stages of plant development and extend into all developmental stages. Germination responds to diurnally fluctuating temperatures in many plant species (Thompson *et al*., 1977). Hypocotyl development is mediated through auxin‐ and temperature‐related processes via the clock‐controlled *PHYTOCHROME‐INTERACTING FACTORS 4* and *5* (*PIF4* and *PIF5*) (Nozue *et al*., 2007; Seaton *et al*., 2015). Many of the central integrators that control flowering time are diurnally expressed, such as *FLOWERING LOCUS T* (*FT*), *SUPPRESSOR OF OVEREXPRESSION OF CO1* (*SOC1*), and *LEAFY* (Wendell *et al*., 2017). Additionally, many of the transcription factors that regulate the synthesis of key plant hormones involved in development, like auxin and abscisic acid (ABA), are diurnally expressed (Balcerowicz *et al*., 2021).

Tissue specificity also plays a role in the link between the clock and development. For instance, the vascular clock plays a dominant role over the epidermal clock in leaves. Moreover, these tissue‐specific clocks influence two distinct developmental processes, flowering and hypocotyl development (Endo *et al*., 2014). Vong *et al*. (2024) suggested that each cell type's transcriptional activity varied across diel and developmental timescales, but it is unclear how the daily oscillations in cell‐type activity vary over developmental timescales.

There remains a large gap in our knowledge of how diel genes vary over development and how developmental genes vary across the day. Here, we address this gap by measuring diel gene expression over one of the most crucial developmental transitions an annual plant experiences: the vegetative‐to‐reproductive transition. We measure gene expression over two timescales: every 24 h (the diel timescale) and over *c*. 2 wk (the developmental timescale). As individual plants experience developmental asynchrony in their floral transition (Klingenberg, 2019; Redmond *et al*., 2024), we use a photoperiod shift from short days (SD; 8 h light d^−1^) to long days (LD; 16 h light d^−1^) to induce synchronised vegetative‐to‐reproductive transitions. One day of LD conditions is sufficient to induce the maximum gene expression response of *FT* (Corbesier *et al*., 1996; Krzymuski *et al*., 2015). We therefore measure diel gene expression in ageing plants after the inductive SD to LD signal.

Here, we determine the extent of transcriptional changes over both developmental and diel timescales. First, we find that gene expression changes most dramatically over the developmental timescale and that core clock genes have broadly stable phases and amplitudes of expression per day. Second, we observe that the expression dynamics of targets of a key Arabidopsis energy sensor vary across both scales. Third, we show that transcriptional targets of core clock genes exhibit differing changes in amplitude over development. Fourth, we identify tissue‐specific changes in rhythmic processes in ageing leaves. Finally, one of the most important applications of our work is in identifying sets of genes that are stable across both timescales, as these could serve as important controls for reverse transcription quantitative polymerase chain reaction. We suggest a filtered set of housekeepers that we would encourage the community to use for studies that aim to study gene expression of clock‐controlled processes in ageing plants.

## Materials and Methods

### Plant growth conditions

**Fig. 1:**
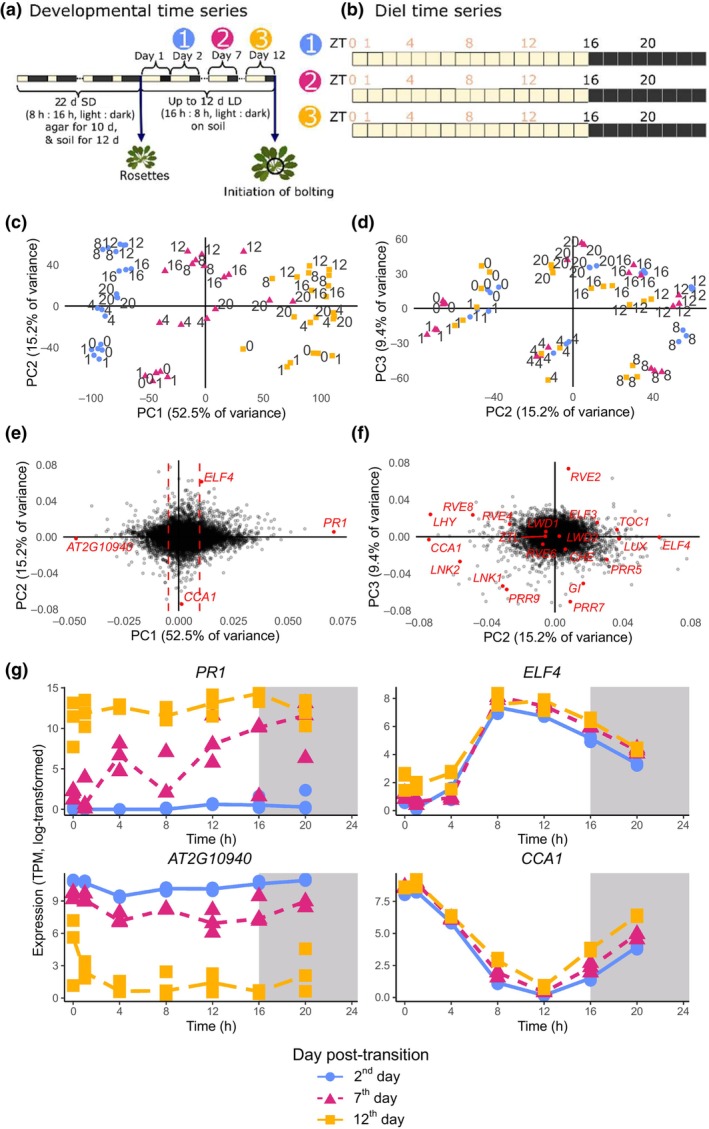
Gene expression dynamics over diel and developmental timescales in *Arabidopsis thaliana*. (a) Samples were collected across three developmental points, following from a short day (SD) to long day (LD) inductive treatment. (b) On each day, plants were sampled at seven diel time points, referred to by their Zeitgeber (ZT) time from dawn. (c) Every individual replicate is plotted on the first two principal components (PCs), using log‐transformed gene expression as inputs. The labelled numbers represent the time of day relative to dawn (ZT). Colour and shape represent the day of sample collection, according to the legend at the bottom. (d) The same as (c) but for the second and third PCs. (e) The distribution of genes according to the loadings on PC1 and PC2. Dashed vertical lines represent the 10% and 90% percentiles on PC1. Genes highlighted in red are plotted in (g). (f) The distribution of genes according to the loadings on PC2 and PC3. Core clock genes (as defined by Wang *et al*., 2024) are highlighted in red. (g) Expression of the genes with the most positive and negative loadings on PC1 and two core clock genes, *CIRCADIAN CLOCK ASSOCIATED1* (*CCA1*) and *EARLY FLOWERING4* (*ELF4*). The *x*‐axis represents ZT on each day. Points represent individual biological replicates. Lines are drawn through the median value of the biological replicates. There are three replicates per time point, but some of the replicates have very similar expression values, and so the points appear to overlap each other. *CHE*, *CCA1 HIKING EXPEDITION*; *GI*, *GIGANTEA*; *LNK1*, *NIGHT LIGHT INDUCIBLE1*; *LNK2*, *NIGHT LIGHT INDUCIBLE2*; *LHY*, *LATE ELONGATED HYPOCOTYL*; *LUX, LUX ARRHYTHMO*; *LWD1*, *LIGHT‐REGULATED WD1*; *LWD2*, *LIGHT‐REGULATED WD2*; *PR1*, *PATHOGENESIS‐RELATED GENE 1*; *PRR5*, *PSEUDO‐RESPONSE REGULATOR5*; *PRR7*, *PSEUDO‐RESPONSE REGULATOR7*; *PRR9*, *PSEUDO‐RESPONSE REGULATOR9*; *TOC 1*, *TIMING OF CAB EXPRESSION 1*; TPM, transcripts per million; *ZTL*, *ZEITLUPE*.

Seeds from the *Arabidopsis thaliana* (L.) Heynh, ecotype Wassilewskija (Ws‐2) (Anwer *et al*., 2020) were surface‐sterilised. They were then plated onto 1× Murashige & Skoog basal salts (Duchefa Biochemie, Haarlem, the Netherlands) supplemented with 1% w/v sucrose (ThermoFisher, Waltham, MA, USA), 0.5% w/v MES (Melford Bioscience, Ipswich, UK), and 1.5% w/v phytoagar (Duchefa Biochemie). After 4 d of stratification at 4°C, these plates were then transferred to SD conditions (8 h : 16 h, light : dark; 70 μmol m^−2^ s^−1^ with vertical fluorescent lighting; Osram L 36 W/830 Warm White) at 21°C and left for 10 d. Then, two seedlings per pot were transferred to soil and thinned out to one seedling per pot after 3 d. Third and fourth true rosette leaves were tracked for each individual plant by placing pipette tips in soil next to these leaves and adjusting them as the leaves moved. However, 12 d after transferring to soil, the conditions were changed to LD (16 h : 8 h, light : dark) at 21°C, as shown in Fig. 1(a).

Samples were collected at ZT (zeitgeiber time; hours after dawn) 0, 1, 4, 8, 12, 16, 20 on 3 d, as shown in Fig. 1(b). We chose to include the ZT1 timepoint due to our previous observations that gene expression changes rapidly in the first hour after dawn (Balcerowicz *et al*., 2021). The first day of sample collection started 24 h after ZT0 of the first LD (2^nd^‐day post‐transition). The second day started 5 d later (7^th^‐day post‐transition). The third started another 5 d later (12^th^‐day post‐transition). Three replicates for each time point were taken – with each replicate consisting of the 3^rd^ and 4^th^ true rosette leaves of two separate plants. These were flash‐frozen in liquid nitrogen and then stored at −80°C. For collections during dark conditions (i.e. ZT16 and ZT20), this work was carried out under green‐filtered light.

### RNA‐seq and initial data processing

RNA was extracted using the Monarch Total RNA Miniprep kit (New England Biolabs, Ipswich, MA, USA), according to the manufacturer's protocol. Residual genomic DNA treatment was removed using the Invitrogen TURBO DNA‐free kit (ThermoFisher), using ‘Routine DNase treatment’, according to the manufacturer's protocol.

Libraries were prepared from high‐quality RNA by the Genomics Laboratory of the University of York's Bioscience Technology Facility. Libraries were prepared using the NEBNext Ultra II Directional Library prep kit for Illumina in conjunction with the NEBNext poly(A) magnetic isolation module (New England Biolabs). Library quality was assessed using the Agilent 2100 Bioanalyzer instrument (Agilent Techologies, Santa Clara, CA, USA) before pooling at equimolar ratios. Pooled libraries were sent for paired‐end 150‐base sequencing by Novogene (UK) Ltd (Cambridge, UK), on an Illumina NovaSeq 6000.

Before further processing of the raw sequencing data, FastQC v.0.11.7 (Andrews, 2010) was used to ensure sample quality passed the expected checks for RNA‐seq data. Illumina adapters and low‐quality bases at the 3′ and 5′ ends were trimmed using Cutadapt v.3.4 (Martin, 2011). Reads were quantified using Salmon v.1.6.0 (Patro *et al*., 2017) against the TAIR10 transcriptome (Berardini *et al*., 2015).

R (v.4.2.3) was used for analysis of gene expression data (R Core Team, 2023). Transcripts per million (TPM) was used as the measure of relative gene expression across samples. Additionally, TPM levels per transcript isoform were combined to leave only gene‐level expression data. In this study, 13 156 genes were filtered out before further analysis if all median TPM values (across the three biological replicates per time of day and sampling day) were ≤ 0.5 (Supporting Information Fig. S1; Table S1). Where log‐transformed gene expression is referred to in this text, the transformed expression is log_2_(TPM + 1).

### Principal component analysis and gene ontology term overrepresentation

Principal component analysis (PCA) was performed using ‘prcomp’ on log‐transformed gene expression on all samples. All gene ontology (GO) term overrepresentation was performed using the gprofiler2 R package (v.0.2.2) (Kolberg *et al*., 2020). This called the g:Profiler server (v.e111_eg58_p18_f463989d), which utilises the Set Counts and Sizes (g:SCS) multiple testing correction method, and we then applied a significance threshold of 0.05 (Kolberg *et al*., 2023) (Tables S2, S3, S6, to be described later). Related groups of genes were submitted as ‘multi queries’.

### CIBERSORTx

Transcripts per million tables were input into the cell fraction mode of the CIBERSORTx web interface (https://cibersortx.stanford.edu/, accessed 28 May 2024), using 100 permutations for the permutation test to assess the *P*‐values (Newman *et al*., 2019). The signature matrix came from Vong *et al*. (2024) and was trained on the single‐cell RNA‐seq data from Procko *et al*. (2022).

### Rhythmic gene prediction

To predict rhythmic genes, we applied the R package metacycle (v.1.2.0) to log‐transformed gene expression, only allowing the JTK_cycle method (Hughes *et al*., 2010; Wu *et al*., 2016). Samples at ZT1 were excluded since JTK_cycle required evenly spaced time points. Genes were classified as rhythmic if and only if the ‘BH.Q’ value was < 0.05. The same method was used to classify the rhythmicity of cell types, where instead we used unscaled cell proportions as inputs.

### Data from previous publications

To identify the putative targets of *SNF1 KINASE HOMOLOG10* (*KIN10*), we took the ‘AGI number’ column from each of the ‘INCREASED’ and ‘DECREASED’ sections from table S3 of Baena‐González *et al*. (2007). Putative targets of *CCA1* were collected from dataset S2 of Nagel *et al*. (2015). Putative targets of *EARLY FLOWERING3* (*ELF3*) were collected from table S6 of Ezer *et al*. (2017). Housekeeping genes suggested for developmental and diel time series were collected from table S1 of Czechowski *et al*. (2005). Only gene names that are consistent with our processed RNA‐seq were used.

## Results

### Gene expression varies more across development than across a diel cycle

Since the clock is tightly linked to changes in gene expression over plant development, we first sought to determine whether diel or developmental time had a greater impact on gene expression.

We measured gene expression across both timescales, as shown in Fig. 1(a,b), performing RNA‐seq across 3 d after a photoperiod transition, which was used to induce synchronised bolting. Previous work has demonstrated that lengthening the photoperiod can serve as an inductive signal to flower early (Cookson *et al*., 2007; Del Prete *et al*., 2019). The Ws‐2 plants are at a rosette phase during the photoperiod transition. The leaves increase in size and number after the photoperiod transition, with no individual plants undergoing bolting on days 2 and 7. Previous research suggests that developmental processes and growth are arrested in leaves 3 and 4 (the leaves sampled) following the photoperiod transition (Del Prete *et al*., 2019). However, all leaves became less round and more elongated and serrated between the photoperiod shift and day 7. On day 12, individuals did not display a 1 cm long bolt (our definition of bolting in Redmond *et al*., 2024), but for some of the population there were visible signs of the initiation of the bolting process. There was no visual evidence of senescence (i.e. no visible yellowing). However, our previous work suggests that there may be early transcriptional markers of senescence in bolting plants without visible signs of senescence (Redmond *et al*., 2024).

Principal component analysis shows that the first principal component (PC) primarily separates out samples based on developmental time, suggesting that variation in gene expression across development dominates variation from diel oscillations (Fig. 1c). As expected, the central day (7^th^ day after transition) lies in between the early (2^nd^) and late (12^th^) days. This agrees with clustering based on the correlation between samples (Fig. S2). Moreover, 52.5% of variance within the filtered dataset can be explained by the developmental‐related PC – indicating a large transcriptomic scale shift in the 12 d after the transition (Figs 1c, S3).

*CONSTANS* forms a complex with nuclear factor Y (NF‐Y), binding DNA and regulating FT, a key flowering time gene (Duplat‐Bermúdez *et al*., 2016; Gnesutta *et al*., 2017). To confirm that the molecular pathway to induce flowering is consistent with the literature, we monitored the expression of genes that were found to be downstream of the NF‐Y/CO complex (Gnesutta *et al*., 2017). We found that most genes that increased their expression in mutants of the NF‐Y/CO complex (specifically *co‐sail, nf‐yb2 nf‐yb3*, and *nf‐yc3 nfyc4 nfyc9*) showed highest expression on 12‐d post‐shift (Fig. S4). About a third of genes that had reduced expression in these mutants had highest expression on day 2, whereas another third had consistent expression over the time series but were primarily expressed at dawn, a time point where there is a rapid induction of expression (Balcerowicz *et al*., 2021). In the next two principal components (PC2 and PC3), samples clustered based on time of day, not day of sampling, with samples forming a circle ordered by the time of day that samples were collected (Fig. 1d). For example, *LATE ELONGATED HYPOCOTYL* and *CCA1* are morning genes (Alabadí *et al*., 2001) and are found on the left‐hand side of the PCA plot. Cycling towards the bottom of the PCA plot, we observe that the *PSEUDO‐RESPONSE REGULATORS* (PRRs) are ordered in the same way in which they show waves of expression *in planta*, with *PPR9*, then *PRR7*, and finally *PRR5* (Nakamichi *et al*., 2010). GI is also expressed near midday (Park *et al*., 1999) and is found at the bottom of the PCA plot. Finally, *TIMING OF CAB EXPRESSION 1, ELF3, ELF4*, and *LUX* are expressed at the end of the day, with the latter three forming the Evening Complex (Nusinow *et al*., 2011) and these are found on the right‐hand side of the PCA plot. *REVEILLE2* (RVE2) integrates nocturnal temperature information (James *et al*., 2024) and is found at the top of the PCA plot. These results suggest that PCA successfully isolates the developmental and diel components of gene expression in our samples.

By analysing the relative contribution (i.e. loadings) of each gene to the first three PCs, we can identify genes that may be associated with developmental (PC1) or diel (PC2/3) processes (Fig. 1e,f). For instance, we can observe that the primary circadian clock genes, as defined by (Wang *et al*., 2024), are correctly ordered in the loadings in Fig. 1(f). This includes a precise counter‐clockwise ordering of the *PRR* genes by the order of their expected phases (Webb *et al*., 2019). The gene with the most positive loading on PC1 was *PATHOGENESIS‐RELATED GENE 1* (*PR1*; AT2G14610), a salicylic acid‐responsive gene whose expression is tightly linked to leaf senescence (Zhang *et al*., 2013). The gene with the most negative loading on PC1 was a proline‐rich, cell wall protein AT2G10940 (Duruflé *et al*., 2017). As expected, the expression of *PR1* increases post‐transition and the expression of AT2G10940 decreases post‐transition (Fig. 1g). We identified the ‘core clock’ genes, related to the vegetative‐to‐reproductive transition as defined in Wang *et al*. (2024), with the most positive and negative loadings on PC2. The clock gene with most positive PC2 loading was *EARLY FLOWERING 4* (*ELF4*; AT2G40080), a member of the EC (Nusinow *et al*., 2011; Herrero *et al*., 2012; Ezer *et al*., 2017), which peaked around ZT8 in all days. The clock gene with most negative PC2 loading was *CCA1* (AT2G46830), which peaked in the early morning on all days. Interestingly, the transcript level of *CCA1* did not appear to decrease across developmental time (i.e. as the leaves aged), contrary to previous reports using reverse transcription quantitative polymerase chain reaction (Song *et al*., 2018). These results suggest that we can use the PCA loadings to identify genes that are primarily developmental or diurnally regulated.

### Developmental changes in gene expression are linked to metabolism, senescence, and kinase‐related processes

To determine the broad shifts in gene expression that occur across the developmental timescale, we created two groups of the most early‐ and late‐associated genes. These consisted of genes with the most negative 10% of PC1 loadings (associated with high expression on the 2^nd^ day of sampling) and the most positive 10% (associated with high expression on the 12^th^ day of sampling). Gene Ontology (GO) term overrepresented terms among early‐associated genes include ‘photosynthesis’ (GO:0015979) and ‘generation of precursor metabolites and energy’ (GO:0006091), while those overrepresented among late‐associated genes include ‘defence response’ (GO:0006952) and ‘plant organ senescence’ (GO:0090693). These are consistent with the vegetative‐to‐reproductive transition associated GO terms identified in Hinckley & Brusslan (2020) and Redmond *et al*. (2024) (Table S2).

Kinases have been associated with regulating the floral transition, as well as diurnally phosphorylating proteins, forming a bridge between developmental and diel processes (Tsai & Gazzarrini, 2012; Uhrig *et al*., 2021). Since ‘protein serine/threonine kinase activity’ (GO:0004674) was overrepresented in the top 10% of genes by positive PC1 loading, we visualised the distribution of all genes related to this GO term (Table S2; Fig. S5a). The PC1 (developmental time associated) loadings of these genes were significantly different to 0 (two‐sided *t*‐test, *t* = 17.778, *P*‐value < 2.2e‐16) and the mean value was 4.64e‐4 (Fig. S5a). However, there was a much smaller deviation from zero for the two diel time‐associated PCs (PC2 and PC3). The loadings on PC2 were not significantly different from 0 (two‐sided *t*‐test, *t* = −1.8644, *P*‐value = 0.06272; mean value −4.47e‐4) and the loadings for PC3 were significantly different (two‐sided *t*‐test, *t* = 7.4955, *P*‐value = 2.13e‐13; mean value 1.83e‐3), but with a much lower effect size than PC1. This suggests that expression of kinases primarily varies over the developmental timescale.

The four genes from this category with the highest PC1 loading came from two kinase families. Two of these were *WALL‐ASSOCIATED KINASES* (*WAK1*, *WAK3*), which act as pectin receptors and have roles in defence response and cell expansion (Anderson *et al*., 2001; Kohorn & Kohorn, 2012). A further two were *CYSTEINE‐RICH KINASES* (*CRK4*, *CRK45*). Overexpression of *CRK4* is known to enhance pattern‐triggered immunity (Yeh *et al*., 2015). Each of these genes had the property that the expression on day 2 was consistently low throughout the day and the expression on day 12 is consistently high. However, the expression on day 7 spans the range of expression on days 2 and 12, matching day 2 expression at ZT0 and day 12 expression at ZT20 (Fig. S5b). This could explain the significantly nonzero PC3 loadings of kinases since PC3 may be incorporating some developmental variation as well as diel variation.

### Targets of *SNF1 KINASE HOMOLOG 10* show diel and developmental expression changes

**Fig. 2:**
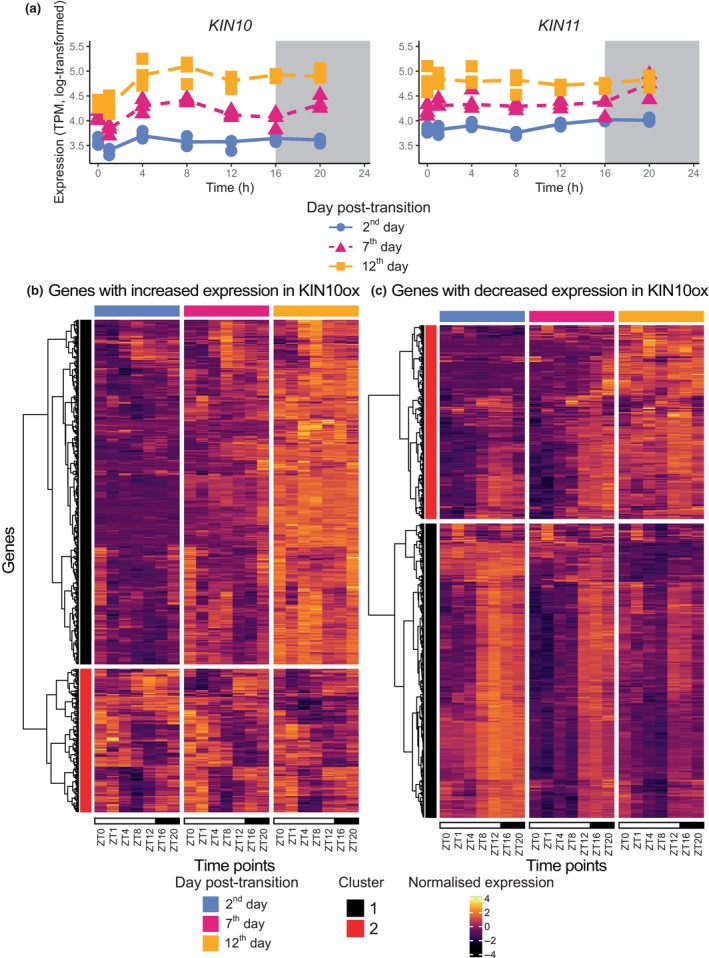
Diel and developmental expression of sucrose nonfermenting‐1‐related protein kinase (SnRK1) components and downstream targets in *Arabidopsis thaliana*. (a) Expression of the two genes that encode the Arabidopsis orthologue of the α subunit of SnRK1, on the same *y*‐axis so they can be compared; the *x*‐axis is Zeitgeber (ZT) time. There are three replicates per time point, but some of the replicates have very similar expression values, and so the points appear to overlap each other. (b) Clustering of genes that showed increased expression in *KINASE10* (*KIN10*) overexpressed Arabidopsis protoplasts, based on normalised gene expression over both timescales. The method ‘ward.D2’ was used to create the hierarchical clustering of the rows. (c) Same as (b) based on genes that showed decreased expression. *KIN11, KINASE11*; TPM, transcripts per million.

Next, we chose to analyse the diel and developmental variation of a well‐known kinase‐containing protein complex that functions as an energy sensor in Arabidopsis: the sucrose nonfermenting‐1‐related protein kinase (SnRK1) complex (Polge & Thomas, 2007; Broeckx *et al*., 2016), which can entrain the clock in response to sugars (Frank *et al*., 2018) and can regulate growth and development in response to sugar status (Chen *et al*., 2017). The SnRK1 complex consists of three subunits, each of which is encoded from different homologues (Broeckx *et al*., 2016). *SNF1 KINASE HOMOLOG10* and *SNF1 KINASE HOMOLOG11* (*KIN11*) are protein serine/threonine kinases, which encode the α subunit of SnRK1. Importantly, homologues of all three subunits may be expressed specifically in different developmental stages and environmental conditions (Broeckx *et al*., 2016). Both *KIN10* and *KIN11* had increased expression over the developmental timescale (Fig. 2a). However, especially on the 7^th^ and 12^th^ day, *KIN10* expression had a larger diel variation than *KIN11*. As for the β subunit, we found that *KINβ1* expression had much larger variation over both the diel and developmental timescales than the expression of its homologues *KINβ2* and *KINβ3* (Fig. S6a). Finally, the essential gene *KINβγ* (*KINbeta‐gamma, HOMOLOG OF YEAST SUCROSE NONFERMENTING 4, SNF4*) was far more stably expressed over development than the related *KINγ* (*KINgamma*) (Fig. S6b; Ramon *et al*., 2013). This could be explained by the fact that *KINγ* is not suspected to form a functional part of the SnRK1 complex, as opposed to its orthologue in mammalian systems (Emanuelle *et al*., 2015).

Since *KIN10* is known to activate and repress a wide range of pathways (Baena‐González *et al*., 2007), we asked how the expression of processes that act downstream of *KIN10* changed over diel and developmental timescales. We selected genes which increased or decreased expression by transient *KIN10* overexpression in protoplasts and clustered them based on expression over both timescales (Fig. 2b,c; Baena‐González *et al*., 2007). Both sets of targets clustered into two large subgroups – characterised by their expression patterns over the developmental timescale. In terms of genes with increased expression in the *KIN10* overexpressed lines, Cluster 1 showed a large increase over age, which agrees with greater *KIN10* expression over age (Fig. 2a,b). GO terms significantly overrepresented in this cluster (and not in Cluster 2) included ‘response to hypoxia’ (GO:0001666), ‘response to abscisic acid’ (GO:0009737), and ‘carbohydrate metabolic process’ (GO:0005975) (Table S3). These represented the range of stress responses *KIN10* is known to affect as well as its control over metabolism (Baena‐González *et al*., 2007). Cluster 2 broadly showed a less dramatic increase in expression over age.

In terms of genes with decreased expression in the *KIN10* overexpressed lines, the two clusters showed strikingly different expression. Cluster 1 expression broadly decreased in expression over age, which agrees with increasing *KIN10* expression (Fig. 2a). The majority of these targets also peaked at ZT12 or later in the day, when starch is degraded to provide a carbohydrate source (Fig. 2c; Streb & Zeeman, 2012). This cluster appeared to represent the sink‐to‐source transition of ageing leaves, since both ‘primary metabolic process’ (GO:0044238) and ‘cellular nitrogen compound metabolic process’ (GO:0034641) were significantly overrepresented (Table S3; Havé *et al*., 2017). Contrarily, Cluster 2 showed a broad increase in expression. Contrary to expectations, this shows that some genes that have reduced expression in the *KIN10* over‐expressor will have higher expression in the developmental stages where *KIN10* is most highly expressed. Our results suggest that SnRK1 sub‐components primarily changed their transcriptional profiles developmentally, but that most genes that were downregulated in response to *KIN10* expression have consistent diel expression patterns.

### Many targets of core clock genes vary their expression patterns over development

Although PCs 2 and 3 (the diel components) accounted for only 24.6% of the variation in our dataset and the diel variation of *KIN10* expression was relatively small, metabolic signals are known to be both inputs and outputs of the circadian clock (Shin *et al*., 2017; Frank *et al*., 2018; Cervela‐Cardona *et al*., 2021). Additionally, senescence‐associated changes in period and phase have been observed in both the core clock genes and the wider circadian transcriptome (Kim *et al*., 2016, 2018; Buckley *et al*., 2024). We noticed that most clock genes had expression patterns that were consistent across development under our fixed long‐day conditions (Fig. S7). This prompts the questions of whether clock gene *targets* had stable expression over development and whether they were rhythmic each day after the SD to LD transition.

**Fig. 3:**
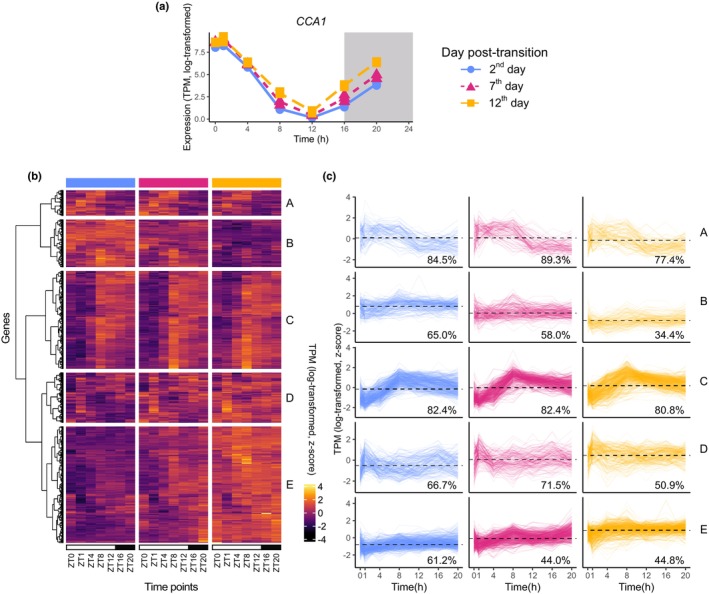
Diel and developmental expression of *CIRCADIAN CLOCK ASSOCIATED1* (*CCA1*) and downstream targets in *Arabidopsis thaliana*. (a) Expression of *CCA1* over Zeitgeber (ZT) time. There are three replicates per time point, but some of the replicates have very similar expression values and so the points appear to overlap each other. (b) Heatmap of targets from *CCA1*, from Chromatin immunoprecipitation sequencing (ChIP‐seq) data in Nagel *et al*. (2015). Values represent log‐transformed, then *z*‐scored gene expression, so the expression can be compared across different genes. (c) Timeseries of targets, grouped by cluster. These follow the same vertical order as in (b). Dashed lines represent mean expression over all genes plotted and all timepoints. The percentages refer to the proportion of genes that were significantly rhythmic in each day, as predicted by JTK_cycle. TPM, transcripts per million.

*CIRCADIAN‐CLOCK ASSOCIATED 1* is a core clock gene whose expression peaks in the early morning under LDs (Fig. 3a; Webb *et al*., 2019; Wang *et al*., 2024). We collected predicted *CCA1* targets from a previous Chromatin immunoprecipitation sequencing (ChIP‐seq) study, where seedlings were grown under 12 h : 12 h, light : dark conditions (Nagel *et al*., 2015). We observed that the median expression of *CCA1*, at every time of day, increased slightly over development (Fig. 3a). Surprisingly, we found that cluster targets of *CCA1* demonstrated increasing, decreasing, and stable mean expression per day of the experiment (Fig. 3b,c; Table S5, to be described later). Further, we found that some clusters of targets became less rhythmic over age (e.g. Clusters B and E), according to JTK_cycle, whereas some clusters maintained high levels (> 80%) of rhythmicity (Cluster C) (Hughes *et al*., 2010).

**Fig. 4:**
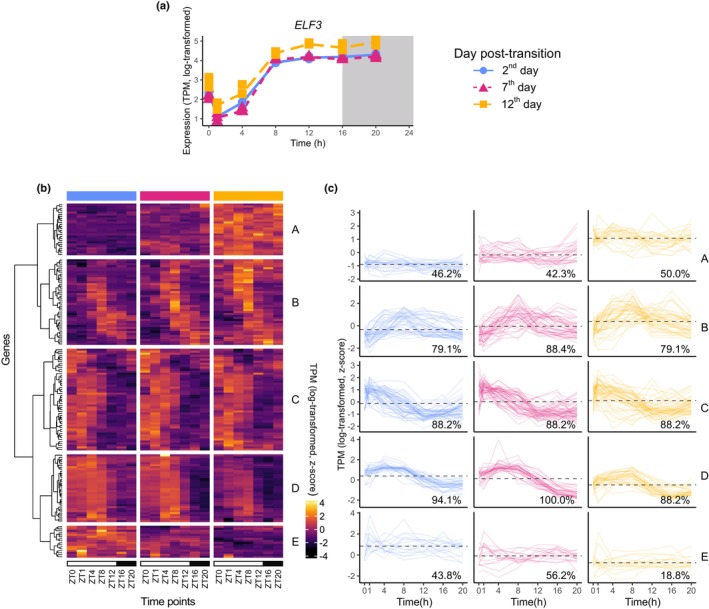
Diel and developmental expression of *EARLY FLOWERING3* (*ELF3*) and downstream targets in *Arabidopsis thaliana*. (a) Expression of *ELF3*, over Zeitgeber (ZT) time. There are three replicates per time point, but some of the replicates have very similar expression values, and so the points appear to overlap each other. (b) Heatmap of targets from *ELF3*, from Chromatin immunoprecipitation sequencing (ChIP‐seq) data in Ezer *et al*. (2017). Values represent log‐transformed then *z*‐scored gene expression, so the expression can be compared across different genes. (c) Timeseries of targets, grouped by cluster. TPM, transcripts per million.

To show if these complicated patterns of regulation were preserved across other clock genes, we looked at predicted targets of another clock gene, *ELF3*, from Ezer *et al*. (2017). *ELF3* is a component of the EC and its expression peaks around ZT8 under LDs, 8 h later than *CCA1* (Fig. 4a; Ezer *et al*., 2017; Webb *et al*., 2019). As for highly rhythmic clusters, they showed varying behaviour over development. Clusters C and D were both highly rhythmic on the 2^nd^, 7^th^, and 12^th^ days and their expression broadly peaked before ZT8 on each day, suggesting that they were repressed by the EC (Fig. 4b,c; Tables S4, S5). The mean expression of Cluster C over each day slightly increased whereas this decreased for Cluster D. Both clusters had an overrepresentation of ‘photosystem I’ (GO:0009522) and multiple other photosynthesis‐related terms (Table S6). However, many regulatory GO terms were overrepresented in Cluster C but not in Cluster D, such as ‘regulation of biosynthetic process’ (GO:0009889) and ‘response to red or far red light’ (GO:0009639). *PHYTOCHROME‐INTERACTING FACTOR5*, which is phosphorylated and degraded in response to red‐light, was in Cluster C (Shen *et al*., 2007). This suggests that the interplay between the EC and *PHYTOCHROME B* (Ezer *et al*., 2017) may be stable over development.

### Cell‐type‐specific activity changes over diel and developmental timescales

Leaves are composed of diverse cell types, such as mesophyll, epidermal, and vascular cells. Our previous work suggests that different cell types may have different relative levels of transcriptional activity at different times of day and different developmental stages (Vong *et al*., 2024). However, it is unclear whether the diel oscillations in cell‐type‐specific transcriptional activity change over development. It is important to perform this type of analysis because differences in expression over time can potentially emerge due to different spatial patterns in expression, caused by different cells being transcriptionally active at different time points.

**Fig. 5:**
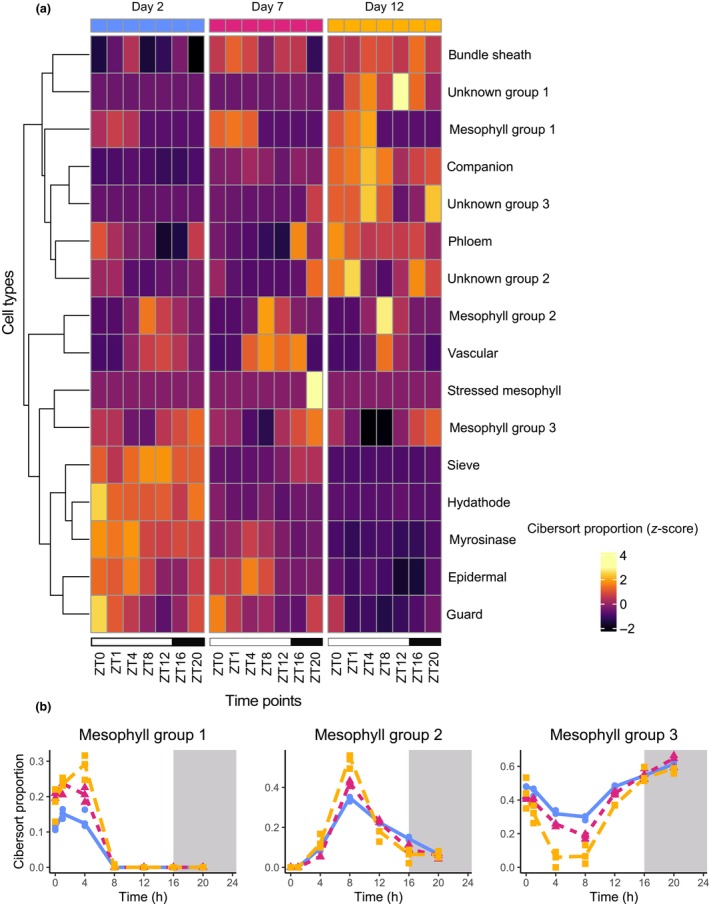
Diel and developmental transcriptional activity by cell type in *Arabidopsis thaliana*. (a) Heatmap of CibersortX outputs. Values represent the predicted proportion of gene expression from each sample that came from each cell type. Values shown represent the median value of the three biological replicates, which have been scaled and then values higher than 3 or lower than −3 have been truncated. Rows have been clustered with hierarchical clustering using the ‘complete’ method. Zeitgeber (ZT) time is shown. (b) The proportion of three different groups of mesophyll cell types. Points represent the values for the three individual replicates and lines are drawn through median values.

Since different clock components are enriched in various Arabidopsis leaf cell types (Nohales, 2021; Davis *et al*., 2022), we wanted to know how cell‐specific diel expression of genes changes over age. Our previous work adapted a method, CIBERSORTx, to estimate cell‐type‐specific activity in bulk leaf RNA‐seq samples (Newman *et al*., 2019; Vong *et al*., 2024). Most cell types are predicted to have rhythmic activity on all 3 d of sampling (JTK_cycle, BH.q value < 0.05; Fig. S8; Tables S7, S8). However, some cell types with low activity early in development are predicted not to be rhythmic, then become rhythmic later in development, such as ‘Unknown Group 3’. Vong *et al*. (2024) suggested that these groups may represent stressed or senescent cell types. Our results also confirm that phloem and the unknown cell groups were expressed late in development, while the epidermal cells were primarily transcriptionally active early in development (Fig. 5a). We also confirm consistent diel oscillations in guard cell and vascular cell transcriptional activity. Previous work suggests that endoreduplication is induced in leaves 3 and 4 after shifting plants to LD conditions (Del Prete *et al*., 2019), occurring between 3 and 5 d after the transition. This suggests that there will be more nuclei per cell in the last 2 d in which samples are collected, which could contribute to the cell‐type‐specific expression profiles.

Coupling between the circadian clocks in mesophyll and vasculature has been demonstrated (Endo *et al*., 2014). We therefore sought to understand the behaviour of subgroups of mesophyll. For instance, there were three groups of mesophyll cells that were each primarily active at different times of day and different stages of development. Our results demonstrate that the phase of their expression is consistent throughout development, but that the peak expression increases in groups 1 and 2 over development and the minimum decreases in group 3 (Fig. 5b). These results highlight that any differentially expressed genes over diel or developmental time may be a consequence of oscillations in which cells are transcriptionally active, rather than a homogenous change of expression across all cell types.

### Known housekeeping genes display variation over both developmental and diel timescales

**Fig. 6:**
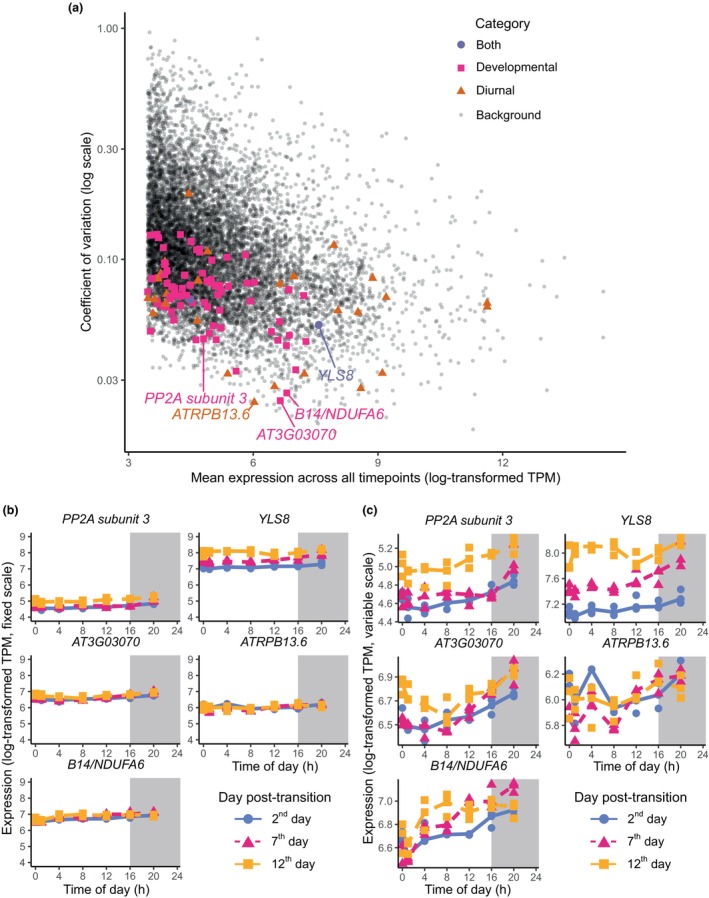
Identification of stably expressed genes over diel and developmental time series in *Arabidopsis thaliana*. (a) Coefficient of variation (CV; calculated by SD/mean) vs mean expression of genes. Both values are calculated including all 63 samples (all replicates at all timepoints). Only genes with transcripts per million (TPM) > 10 are shown. Samples that were suggested by Czechowski *et al*. (2005) as good housekeeping quantitative polymerase chain reaction genes for diurnal experiments or developmental experiments (or both) are highlighted. (b) Expression of five housekeeping genes selected from (a) *PROTEIN PHOSPHOTASE2A* (*PP2A*) subunit 3 and *YELLOW‐LEAF‐SPECIFIC GENE8* (*YLS8*) have relatively high CV values, and the other genes have the lowest three CV values. The *y* axes are fixed so the developmental variation can be compared across genes. (c) Same as (b), except the *y* axes vary to show diurnal variation. There are three replicates per time point, but some of the replicates have very similar expression values, and so the points appear to overlap each other.

Our previous analysis found many examples of genes that change their expression diurnally and/or over development. However, it is also important to find sets of genes that are stably expressed under both conditions. These are important controls in experiments like reverse transcription followed by reverse transcription quantitative polymerase chain reaction, where gene expression must be normalised to a housekeeper gene (Czechowski *et al*., 2005; Souček *et al*., 2017). Researchers often refer to well‐known lists of housekeeping genes, which are suggested to be stable under relevant experimental conditions. We calculated the coefficient of variation (CV; SD divided by mean) from our dataset of the suggested housekeeping genes for developmental and diel experiments from Czechowski *et al*. (2005). A high CV value indicates that a gene shows high biological variability; gene expression is not comparable across different times of day or developmental stages. Although the mean CV value of the housekeeping genes was lower than the mean CV value across all genes, many housekeeping genes still showed undesirable levels of variation (Fig. 6a). For example, two commonly used housekeeping genes, *PROTEIN PHOSPHATASE 2A SUBUNIT3* and *YELLOW‐LEAF‐SPECIFIC GENE8*, had large expression changes over development (Fig. 6b). *YELLOW‐LEAF‐SPECIFIC GENE8* approximately doubled in expression from the 2^nd^ day of sampling to the 12^th^ day. However, some housekeeping genes did show promisingly low CV scores. Genes *ARABIDOPSIS RNA POLYMERASE B13.6* (*ATRPB13.6*, AT3G52090), AT3G03070, and AT3G12260 had the lowest CV values across all housekeeping genes (Fig. 6a). Despite the low CV values, we observed that these genes still displayed variation across the diel timescale when visualised on a relative expression scale (Fig. 6c).

To further confirm that these recommended genes are consistent across experiments, we have demonstrated that they have stable expression in diel and developmental RNA‐seq experiments on leaves that were performed independently by other labs (Fig. S9a,b; Woo *et al*., 2016; Hickman *et al*., 2017). We also show that there is low heterogeneity between leaves from different individuals in a single plant‐omics experiment (Fig. S9c; Redmond *et al*., 2024). Histograms of TPM values of these genes within our dataset are shown in Fig. S9(d–f). We suggest that combinations of these genes may be useful as controls for reverse transcription quantitative polymerase chain reaction.

## Discussion

We measured gene expression via RNA‐seq in ageing plants, across both diel and developmental timescales. Using PCA, we observed that most of the variation in samples can be explained by a developmental PC, with a smaller contribution from two diel PCs (Fig. 1). These PCA loadings help us establish the extent to which any individual gene varies its expression across diel and developmental timescales.

Although kinases are thought to associate diel and developmental programmes, we found that they primarily change their expression across developmental scales. However, many genes whose expression changes are perturbed by *KIN10* overexpression have diel expression patterns, which is consistent with previous suggestions that there is crosstalk between the SnRK1 complex and circadian processes (Chen *et al*., 2017; Shin *et al*., 2017; Frank *et al*., 2018). This highlights that circadian control of primary metabolic processes, including turnover of carbon and nitrogen reserves, remains important during the onset of senescence (Flis *et al*., 2019). Due to the fixed photoperiod that we used throughout the experiment, we could not observe photoperiod‐dependent effects, which have been shown to affect the diel timing of metabolic processes (Flis *et al*., 2016; Alexandre Moraes *et al*., 2022). Future work could test whether these effects are consistent across different stages of plant development.

We did not observe changes in the phase or period of core clock genes (Fig. S7). This is unsurprising given that our ageing plants were exposed to LD (entrainment) conditions on the days of sampling, rather than free‐running conditions, and it highlights the stability of the central oscillator. However, it has previously been observed that the clock can have different free‐running periods in younger vs older leaves (Kim *et al*., 2016). This is consistent with our and previously reported observations that metabolic signalling inputs to the clock, such as *KIN10*, have different levels of expression across plant development (Baena‐González *et al*., 2007; Frank *et al*., 2018).

Despite the relatively stable expression of *CCA1* and *ELF3* over development, their predicted targets cluster into groups that either decrease or increase in mean expression (Figs 3b, 4b). This could be because of changing patterns of core clock gene binding to regulatory regions of the genome. However, these patterns may instead result from downstream processing of circadian signals. For example, the clock influences senescence through both transcriptional and *miR164*‐mediated post‐translation control of *ORESARA1* (*ORE1*) (Kim *et al*., 2018). The extent of these post‐translation mechanisms could be assessed by measuring the changes in the diel proteome and phosphoproteome in ageing plants (Uhrig *et al*., 2021). Our work suggests that there is value in spanning both developmental and diel timescales and suggests that other complementary datasets, such as ChIP‐seq, proteomics, and phosphoproteomics, should be performed at more than one timepoint per day and multiple stages of leaf development (Nagel *et al*., 2015; O'Malley *et al*., 2016; Ezer *et al*., 2017; Li *et al*., 2023).

Diurnal rhythms of gene expression have previously been observed in different cell types, through luciferase assays, reverse transcription quantitative polymerase chain reaction, and single‐nucleus RNA‐seq (Endo *et al*., 2014; Greenwood *et al*., 2022; Qin *et al*., 2023). Here, we utilised CIBERSORTx to deconvolute gene expression from bulk RNA‐seq data into contributions from leaf‐specific cell types, allowing us to see how diel patterns change over development (Newman *et al*., 2019). We identified some cell types that became rhythmic only at the latest stages of development. We also showed that subgroups of mesophyll cells exhibit different timings of expression across both timescales. It remains to be seen whether the reported coordination of the clock across cell types is maintained across development (Endo *et al*., 2014; Gould *et al*., 2018; Schmal *et al*., 2019).

We provide a valuable resource for the community to search for appropriate housekeeping genes when researching the clock in ageing plants. Although existing housekeeping genes show lower variability than background gene expression, we recommend that researchers use at least one of our three suggested genes. Our suggested genes showed little variation across the developmental timescale. Using multiple housekeeping genes to normalise reverse transcription quantitative polymerase chain reaction data may counterbalance the unavoidable diel variation of housekeeping genes (Vandesompele *et al*., 2002).

## Competing interests

None declared.

## Author contributions

EJR was involved in conceptualization, methodology, software, formal analysis, investigation, writing – original draft, and visualization. JR was involved in conceptualization, investigation, and writing – review and editing. SJD was involved in writing – review and editing, supervision, and funding acquisition. DE was involved in conceptualization, methodology, writing – original draft, writing – review and editing, supervision, and funding acquisition.

## Disclaimer

The New Phytologist Foundation remains neutral with regard to jurisdictional claims in maps and in any institutional affiliations.

## Supporting information


**Fig. S1** Determination of filtering threshold.
**Fig. S2** Expression correlations between time points.
**Fig. S3** Percent variance explained by each principal component.
**Fig. S4** Expression of CO‐ and NF‐Y‐related genes.
**Fig. S5** Expression pattern of kinases.
**Fig. S6** Expression pattern of SnRK1 complex components.
**Fig. S7** Expression pattern of core clock genes.
**Fig. S8** Relative transcriptional activity of cell types.
**Fig. S9** Stability of genes over independent RNA‐seq experiments.

**Table S1** Gene expression data in *Arabidopsis thaliana*.

**Table S2** GO overrepresentation of top and bottom 10% genes on PC1 in *Arabidopsis thaliana*.

**Table S3** GO overrepresentation of KIN10 target clusters in *Arabidopsis thaliana*.

**Table S4** Results from JTK_cycle showing which genes are rhythmic in *Arabidopsis thaliana*.

**Table S5** Clusters of CCA1 and ELF3 targets in *Arabidopsis thaliana*.

**Table S6** GO overrepresentation of CCA1 and ELF3 target clusters in *Arabidopsis thaliana*.

**Table S7** Information from CIBERSORTx in *Arabidopsis thaliana*.

**Table S8** Results from JTK_cycle showing which cell types are rhythmic in *Arabidopsis thaliana*.
Please note: Wiley is not responsible for the content or functionality of any Supporting Information supplied by the authors. Any queries (other than missing material) should be directed to the *New Phytologist* Central Office.

## Data Availability

Raw and processed sequencing data have been deposited in the NCBI Gene Expression Omnibus (GEO) database under accession no. GSE242964 (https://www.ncbi.nlm.nih.gov/geo/query/acc.cgi?acc=GSE242964). Scripts used to produce the figures have been deposited in a GitHub repository https://github.com/stressedplants/DiurnalDevelopmental.
